# Comparing cryptocurrencies and gold - a system-GARCH-approach

**DOI:** 10.1007/s40822-022-00218-4

**Published:** 2022-10-05

**Authors:** Jens Klose

**Affiliations:** THM Business School Gießen, Technische Hochschule Mittelhessen, Gießen, Germany

**Keywords:** Cryptocurrencies, Gold, System-GARCH-in-mean, E42, G15, C58

## Abstract

This article investigates similarities and differences between gold and four cryptocurrencies (Bitcoin, Ethereum, Bitcoin Cash and Litecoin) with respect to four determinants. To do so, we estimate a system-GARCH-in-mean for the period starting 7/18/2014 at earliest until 7/12/2021. We find that, first, liquidity premia are almost always insignificant for both gold and cryptocurrencies. Second, volatility premia exist in either gold and cryptocurrencies. Third, the response of cryptocurrencies to exchange rate changes is more pronounced than for gold at least if developing countries are included. Fourth, gold exhibits a safe haven status, while cryptocurrencies do not. So according to our results those cannot be seen as a store of value but rather should be seen as speculative assets.

## Introduction

The use of cryptocurrencies is increasing rapidly in recent years. Because of its independence of national monetary policies and the technology limiting the supply of cryptocurrencies, it may be argued that cryptocurrencies are safe haven assets just like gold. Gold is typically seen as an asset to store value, i.e., in times of turmoil. We will test whether the properties of cryptocurrencies are indeed the same or different from gold.

The literature investigating the role properties of cryptocurrencies is growing rapidly. This article contributes to the literature by estimating a novel system-GARCH in-mean model in this context for four different cryptocurrencies (Bitcoin, Ethereum, Bitcoin Cash and Litecoin), allowing for volatility spillovers among them and comparing those individually to gold. Using a system estimator has the advantage that we can directly compare whether there are significant differences between cryptocurrencies and gold with respect to various determinants. These are: First, an effective exchange rate to cover currency-like properties, second, liquidity premia, third, a measure of global uncertainty and fourth, a volatility premium of the underlying asset and also volatility spillovers from other assets. This being said, this article merges three strands of literature with respect to cryptocurrencies and gold: First, it measures the impact of global uncertainty for cryptocurrencies and gold simultaneously. Second, two new determinants are introduced in the context of cryptocurrencies being a multilateral exchange rate and liquidity. Third, differences between gold and cryptocurrencies with respect to the determinants are investigated empirically.

The results indicate that the negative response to exchange rate is more pronounced for cryptocurrencies than for gold, i.e., if developing countries are added. Liquidity premia are less important in the gold as well as in the cryptocurrency markets. Volatility premia exist for all assets. Cryptocurrencies and gold differ considerably with respect to global uncertainty, i.e., gold can be seen as safe haven asset increasing its value in times of financial stress, the reverse is true with respect to all four cryptocurrencies. So those can be classified as rather speculative assets.

The remainder of the article is organized as follows: Sect. 2 presents a literature review. Section 3 develops the estimation model. Section 4 describes the data used. Section 5 presents the results and Sect. 6 finally concludes.

## Literature review

The economic role of cryptocurrencies has been investigated extensively in recent years in different dimensions. In especially three of those are directly connected to the research question tackled in this article: First, the interconnectedness of different cryptocurrencies. Analysis in this context focuses mainly on volatility spillovers between the different cryptocurrencies.^1^ Corbet et al. (2018) find a strong linkage of Bitcoin, Ripple and Litecoin in prices and volatility but almost no connection to other financial assets. Katsiampa (2018) investigates the volatility interconnectedness of Bitcoin and Ether using a diagonal BEKK model. Indeed a significant degree of interconnectedness of those two cryptocurrencies could be verified. Katsiampa et al. (2019) extend this analysis using a BEKK MGARCH model and adding Litecoin as a third cryptocurrency. They also find strong linkages between those three cryptocurrencies. Andrada-Felix et al. (2020) investigate the role of volatility connectedness of various cryptocurrencies and traditional currencies. They find that connectedness is high between the different cryptocurrencies but they are almost unconnected to traditional currencies.

The second strand of literature investigates the relationship of cryptocurrencies to global uncertainty or crises. Akyildirim et al. (2020) estimate for various cryptocurrencies the correlations with the VIX or VSTOXX. It is shown that there is a correlation, and it increases in times of heightened financial stress. Corbet et al. (2020a) focus on the role of cryptocurrencies in the COVID-19 pandemic. Using social media data, they show that returns as well as volumes traded increase during the pandemic, concluding that cryptocurrencies are a store of value if uncertainty is high. Demir et al. (2020) do a wavelet exercise and focus on COVID-19 cases or deaths instead of social media data. In especially for Bitcoin, a spread of the pandemic reduced the returns on impact but increased them afterwards.

Third, the performance of cryptocurrencies is compared to the one of gold as the classic safe-haven asset in order to find out whether cryptocurrencies exhibit the same properties. Dyhrberg (2016) is one of the first to do so for Bitcoin. He finds out that Bitcoin can be classified in between the US-dollar and gold as the extremes of medium of exchange and store of value. Baur and Dimpfl (2018) replicate and extent the study and come to the result that Bitcoin returns volatility are distinctly different from gold as well as the US-dollar. Corbet et al. (2020b) investigate correlations of Chinese stock markets to either Bitcoin and gold. At least if high-frequency data is used, the correlation of both to stock prices is increasing in the COVID-19 pandemic and therefore possibly also between Bitcoin and gold itself. Zhang and Wang (2021) estimate the effects of financial stress in the US and China on Bitcoin and gold using a volatility connectedness framework in the time-frequency domain. They find that volatility spillovers are relatively high during short-term horizons and increase substantially in uncertain times. Finally, Katsiampa et al. (2019) use a DCC-GJR-GARCH model to estimate the cryptocurrency uncertainty on precious metals. They find that only gold has a consistent and reliable safe-haven status in this setting.

This article merges all three strands of literature, thus we estimate empirically volatility spillovers of four different cryptocurrencies (Bitcoin, Ethereum, Bitcoin Cash and Litecoin), the role of global uncertainty and the differences to gold simultaneously. Moreover, we add two more variables which to the best of our knowledge have not been investigated so far with respect to cryptocurrencies: First, the role of liquidity and second, the impact of a multilateral exchange rate. To do so, we estimate a system-GARCH-in-mean model, which additionally allows us to find significant differences between cryptocurrencies and gold. Closest to this approach is possibly Liu and Serletis (2019) who estimate a VARMA GARCH-in-mean model with respect to volatility and stock prices or interest rates. Our set of variables will be different but we will also allow for volatility spillovers among the different cryptocurrencies and gold.

## The model

The model used in this article rests on a standard portfolio model, according to which certain determinants influence the price of an asset. This can be written as:

1 $$\begin{aligned} p_t = \alpha \cdot ex_t+\beta \cdot l_t+\gamma \cdot r_t+ \delta \cdot v_t \end{aligned}$$

In eq. (1), the asset price ($p_t$) is either the price of gold or a cryptocurrency. Four determinants explain the price level while $\alpha , \beta , \gamma$ and $\delta$ determine the quantitative effect of those on asset prices: First, assets respond to fundamental factors. Since cryptocurrencies, as well as gold, should have currency-like properties, they should vary with some kind of exchange rate ($ex_t$), i.e., the prices should rise if the underlying currency is depreciating and vice versa.

Second, the price of an asset is driven by its liquidity ($l_t$), meaning that investors demand a higher liquidity premium the more illiquid the asset. Thus, prices should be higher for more illiquid assets all else being equal.

Third, assets are influenced by global uncertainty ($r_t$). On the one hand, investors could demand an additional premium if the assets are presumed to be less safe if global uncertainties rise. On the other hand, also a discount is possible for assets viewed as safe havens in crisis periods as it should be the case for gold.

Fourth, a volatility premium is demanded by investors, meaning that assets with higher volatility ($v_t$) need to come up with higher prices all else being equal in order to compensate the investors for the higher uncertainty in future returns. Volatility premia in high-frequency data are typically modeled by adding a GARCH-in-mean term Bollerslev (1986) to the equation (see, e.g., Katsiampa et al. 2019). So as an econometric specification the volatility premium in eq. (1) can be substituted by the GARCH-in-mean term:

2 $$\begin{aligned} p_t = \alpha \cdot ex_t+\beta \cdot l_t+\gamma \cdot r_t+ \delta \cdot \sigma _t^2+\varepsilon _t \end{aligned}$$

In eq. (2) $\sigma _t^2$ signals the GARCH term and $\varepsilon _t$ the residuals. In line with the literature on cryptocurrency estimation, we use a standard GARCH (1,1) model (see Dyhrberg 2016; Baur and Dimpfl 2018; Corbet et al. 2020a, b; Akyildirim et al. 2020):

3 $$\begin{aligned} \sigma _t^2 = \zeta + \eta \cdot \varepsilon _{t-1}^2 + \theta \cdot \sigma _{t-1}^2 \end{aligned}$$

However, we want to estimate eqs. (2) and (3) not only for just one asset but for five different assets being gold as well as the four cryptocurrencies Bitcoin, Ethereum, Bitcoin Cash and Litecoin. This being said, we do not only account for the volatility of each asset separately but allow for cross-correlation of the various assets. This leads to the following to system:

4 $$\begin{aligned} \mathbf {p}_{\mathbf{t}} = \mathbf{a} \,\mathbf{ex}_{\mathbf{t}} + \mathbf{L}_{\mathbf{t}} \, \mathbf{b} + \mathbf{c}\, \mathbf{r}_{\mathbf{t}} + \mathbf{s}'_{\mathbf{t}} \, \mathbf{D} \, \mathbf{s}_{\mathbf{t}}+ \mathbf{e}_{\mathbf{t}}, \end{aligned}$$

with $${\mathbf {a}} = \begin{pmatrix} \alpha _1 \\ \alpha _2 \\ \alpha _3 \\ \alpha _4 \\ \alpha _5 \end{pmatrix}$$, $${\mathbf {b}} = \begin{pmatrix} \beta _1 \\ \beta _2 \\ \beta _3 \\ \beta _4 \\ \beta _5 \end{pmatrix}$$, $${\mathbf {c}} = \begin{pmatrix} \gamma _1 \\ \gamma _2 \\ \gamma _3 \\ \gamma _4 \\ \gamma _5 \end{pmatrix}$$, $$\mathbf {p_t} = \begin{pmatrix} p_{1t} \\ p_{2t} \\ p_{3t} \\ p_{4t} \\ p_{5t} \end{pmatrix}$$, $$\mathbf {s_t} = \begin{pmatrix} \sigma _{1t} \\ \sigma _{2t} \\ \sigma _{3t} \\ \sigma _{4t} \\ \sigma _{5t} \end{pmatrix}$$,

$$\begin{aligned}\mathbf {e_t} =& \begin{pmatrix} \varepsilon _{1t} \\ \varepsilon _{2t} \\ \varepsilon _{3t} \\ \varepsilon _{4t} \\ \varepsilon _{5t} \end{pmatrix}, \mathbf {ex_t} = \begin{pmatrix} ex_t \end{pmatrix}, \mathbf {r_t} = \begin{pmatrix} r_t \end{pmatrix}, {\mathbf {L}} =\begin{pmatrix} l_{1t} &{} 0 &{} 0 &{} 0 &{} 0 \\ 0 &{} l_{2t} &{} 0 &{} 0 &{} 0 \\ 0 &{} 0 &{} l_{3t} &{} 0 &{} 0 \\ 0 &{} 0 &{} 0 &{} l_{4t} &{} 0 \\ 0 &{} 0 &{} 0 &{} 0 &{} l_{5t} \\ \end{pmatrix},\\ {\mathbf {D}}=& {} \begin{pmatrix} \delta _{11} &{} \delta _{12} &{} \delta _{13} &{} \delta _{14} &{} \delta _{15} \\ \delta _{21} &{} \delta _{22} &{} \delta _{23} &{} \delta _{24} &{} \delta _{25} \\ \delta _{31} &{} \delta _{32} &{} \delta _{33} &{} \delta _{34} &{} \delta _{35} \\ \delta _{41} &{} \delta _{42} &{} \delta _{43} &{} \delta _{44} &{} \delta _{45} \\ \delta _{51} &{} \delta _{52} &{} \delta _{53} &{} \delta _{54} &{} \delta _{55} \\ \end{pmatrix}.\end{aligned}$$

Please note, that $ex_t$ and $r_t$ are simple scalars as those variables are the same for all assets.

The variances and covariances are estimated using a diagonal-BEKK-GARCH Engle and Kroner (1995). Thus, those have the following form:

5 $$\begin{aligned} {\mathbf{S}}_{\mathbf{ij} \, \mathbf{t}} = \mathbf{F}' \, \mathbf{F} + \mathbf{G}' \, \mathbf{e}_{\mathbf{t}-\mathbf{1}} \, \mathbf{e}'_{\mathbf{t}-\mathbf{1}} \, \mathbf{G}+ \mathbf{H}' \, \mathbf{S}_{\mathbf{ij} \, \mathbf{t}-\mathbf{1}} \, \mathbf{H}, \end{aligned}$$

with

$$\begin{aligned} {\mathbf {S}}_{\mathbf{ij} \, \mathbf{t}} =&\begin{pmatrix} \sigma _{1t}^2 &{} \sigma _{1t}\cdot \sigma _{2t} &{} \sigma _{1t}\cdot \sigma _{3t} &{} \sigma _{1t}\cdot \sigma _{4t} &{} \sigma _{1t}\cdot \sigma _{5t} \\ \sigma _{1t}\cdot \sigma _{2t} &{} \sigma _{2t}^2 &{} \sigma _{2t}\cdot \sigma _{3t} &{} \sigma _{2t}\cdot \sigma _{4t} &{} \sigma _{2t}\cdot \sigma _{5t} \\ \sigma _{1t}\cdot \sigma _{3t} &{} \sigma _{2t}\cdot \sigma _{3t} &{} \sigma _{3t}^2 &{} \sigma _{3t}\cdot \sigma _{4t} &{} \sigma _{3t}\cdot \sigma _{5t} \\ \sigma _{1t}\cdot \sigma _{4t} &{} \sigma _{2t}\cdot \sigma _{4t} &{} \sigma _{3t}\cdot \sigma _{4t} &{} \sigma _{4t}^2 &{} \sigma _{4t}\cdot \sigma _{5t} \\ \sigma _{1t}\cdot \sigma _{5t} &{} \sigma _{2t}\cdot \sigma _{5t} &{} \sigma _{3t}\cdot \sigma _{5t} &{} \sigma _{4t}\cdot \sigma _{5t} &{} \sigma _{5t}^2 \\ \end{pmatrix},\\ {\mathbf {F}}= & {} \begin{pmatrix} \zeta _{11} &{} \zeta _{12} &{} \zeta _{13} &{} \zeta _{14} &{} \zeta _{15} \\ 0 &{} \zeta _{22} &{} \zeta _{23} &{} \zeta _{24} &{} \zeta _{25} \\ 0 &{} 0 &{} \zeta _{33} &{} \zeta _{34} &{} \zeta _{35} \\ 0 &{} 0 &{} 0 &{} \zeta _{44} &{} \zeta _{45} \\ 0 &{} 0 &{} 0 &{} 0 &{} \zeta _{55} \\ \end{pmatrix}, {\mathbf {G}} =\begin{pmatrix} \eta _{11} &{} 0 &{} 0 &{} 0 &{} 0 \\ 0 &{} \eta _{22} &{} 0 &{} 0 &{} 0 \\ 0 &{} 0 &{} \eta _{33} &{} 0 &{} 0 \\ 0 &{} 0 &{} 0 &{} \eta _{44} &{} 0 \\ 0 &{} 0 &{} 0 &{} 0 &{} \eta _{55} \\ \end{pmatrix},\\ {\mathbf {H}}= & {} \begin{pmatrix} \theta _{11} &{} 0 &{} 0 &{} 0 &{} 0 \\ 0 &{} \theta _{22} &{} 0 &{} 0 &{} 0 \\ 0 &{} 0 &{} \theta _{33} &{} 0 &{} 0 \\ 0 &{} 0 &{} 0 &{} \theta _{44} &{} 0 \\ 0 &{} 0 &{} 0 &{} 0 &{} \theta _{55} \\ \end{pmatrix}.\end{aligned}$$

## Data

In this section, we describe the data used. Throughout the article, we make use of daily data, excluding weekends and holidays. Given the model of the previous section, we have five dependent variables in our system. These are the prices for gold, Bitcoin, Ethereum, Bitcoin Cash and Litecoin. In order to compare the prices, they are all denominated in US-dollar. Even though gold and cryptocurrencies are traded in different currencies, we use the US-dollar as the world leading currency in order to rely on a comparable set of variables. I.e., all other explanatory variables are also modeled in US-dollar terms or in case of the exchange rate against the US-dollar.

The four cryptocurrencies investigated are chosen because of their relative importance in the cryptocurrency market as measured by their market capitalization. Moreover, we implement two further restrictions on cryptocurrencies to be considered in this analysis. First, cryptocurrencies have to be traded actively for quite some time in order to generate enough data points to be able to estimate meaningful results. This is e.g., the reason why Dogecoin or Solana have not been included. Second, stablecoins like Tether are excluded from the analysis as those by definition are fixed to some currency i.e., the US-dollar. As of July 2021 (the end of our sample period) the share in market capitalization of our four cryptocurrencies in overall cyrptocurrency market including stablecoins is: Bitcoin 43.69%, Ethereum 18.10%, Litecoin 1.09% and Bitcoin Cash 0.89%.

The prices are collected together with the liquidity indicator for those five assets. As frequently used in the literature, liquidity is measured by the underlying bid-ask-spread of the asset (see, e.g., Bernoth and Erdogan 2012; Afonso et al. 2015 or Klose 2021). Thus, a higher bid-ask spread signals lower liquidity of the asset, which should lead to a higher liquidity premium demanded by the investors. The availability of the various bid-ask spreads is determining our sample period. For cryptocurrencies there are no corresponding long-term data. Thus, the first data are available for Bitcoin on 7/18/2014, for Bitcoin Cash on 2/12/2018 for Ethereum and Litecoin on 11/01/2018. The end of the sample is for all assets 7/12/2021.

As the asset prices are denominated in US-dollar, this is also the one currency the exchange rate is based on. However, it makes no sense to use a bilateral exchange rate, e.g., towards the Euro, as this may not represent the overall evolution of the US-dollar. Therefore, we use effective exchange rates. Those are used in nominal terms as also the asset prices are nominal. The Bank of International Settlements (BIS) collects two types of daily effective exchange rates. The first one is a narrow effective exchange rate towards 24 other economies.^2^ The second one is a broad effective exchange rate covering the 24 economies of the narrow aggregate and adding 34 other economies.^3^ So in total 58 economies are covered by the broad effective exchange rate. We will use both effective exchange rate as there may be differences between the country samples, i.e., as the broad effective exchange rates adds especially developing and transition countries which may behave differently from developed countries when it comes to the use of cryptocurrencies.

To cover global uncertainty, two variables are frequently used: The first one is the volatility index (VIX) covering the implied volatility of the S&P 500. Thus, higher volatility signals a higher degree of global stress. The second one is the US corporate BBB government bond spread.^4^ A rising spread is here the indicator for increasing global uncertainty. We will use both measures but expect the qualitatively same influence on our asset prices.

Please note, that there are no data collected for the (cross-) volatility, as these variables are estimated within the system via GARCH.

**Table 1:** Descriptive statistics and stationarity tests

	Mean	Std. Dev.	Skewness	Kurtosis	Jarque-Bera	ADF	PP
*Level*							
Gold price	1370.97	230.54	1.22	3.32	498.52***	−0.51	−0.49
Bitcoin price	7840.06	11925.53	2.75	10.56	6632.04***	−0.78	−0.61
Ethereum price	596.91	800.59	2.05	6.45	842.24***	−0.71	−0.72
Bitcoin cash price	444.21	310.65	1.62	5.27	582.82***	−3.47***	−3.00**
Litecoin price	86.79	63.03	1.85	6.41	741.42***	−2.14	−1.76
Gold bid-ask-spread	2.20	2.07	3.42	35.22	88750.80***	−2.27	−27.94***
Bitcoin bid-ask-spread	4.79	11.96	11.31	193.33	2790631.52***	−10.17***	−40.96***
Ethereum bid-ask-spread	0.32	0.48	0.72	19.43	7969.76***	−3.57***	−21.87***
Bitcoin cash bid-ask-spread	1.08	1.22	3.94	29.30	27987.40***	−5.49***	−17.03***
Litecoin bid-ask-spread	0.11	0.09	1.79	7.29	916.55***	−6.20***	−26.22***
Broad effective exchange rate	117.55	7.23	−1.06	3.65	401.80***	−2.38	−2.38
Narrow effective exchange rate	115.17	5.88	−1.22	3.93	555.65***	−2.53	−2.51
VIX	17.24	7.60	3.10	18.49	22764.85***	−4.83***	−5.17***
US-BBB-government-bond-spread	1.72	0.45	2.13	10.86	6533.46***	−3.74***	−3.23**
*Growth rate*							
Gold price	0.02	0.85	0.03	7.26	1482.12***	−43.25***	−43.24***
Bitcoin price	0.32	4.59	0.31	12.51	6893.28***	−42.52***	−42.54***
Ethereum price	0.52	6.05	0.29	8.29	828.89***	−17.26***	−27.31***
Bitcoin cash price	0.17	7.52	0.98	12.72	3647.63***	−29.31***	−20.45***
Litecoin price	0.35	6.43	0.14	7.37	561.80***	−27.09***	−27.08***
Gold bid-ask-spread	10.15	71.77	10.18	206.95	3434361.76***	−14.88***	−52.00***
Bitcoin bid-ask-spread	2821.19	20638.25	12.51	182.55	2495049.56***	−43.49***	−44.06***
Ethereum bid-ask-spread	378.03	1458.00	5.42	41.84	47555.10***	−7.93***	−29.13***
Bitcoin cash bid-ask-spread	245.50	2769.30	13.73	204.49	1533415.76***	−30.15***	−30.15***
Litecoin bid-ask-spread	114.58	416.53	5.57	41.31	46574.68***	−13.16***	−30.85***
Broad effective exchange rate	0.01	0.31	0.07	5.73	610.44***	−43.91***	−43.91***
Narrow effective exchange rate	0.01	0.36	−0.06	6.19	832.70***	−43.00***	−43.03***
VIX	0.34	8.65	2.62	24.55	40189.22***	−46.35***	−47.48***
US-BBB-government-bond-spread	−0.01	1.34	5.97	105.37	868284.24***	−9.63***	−32.42***

*Notes*: Sample period: varying between 7/18/2014 and 7/12/2021

*Std. Dev.* Standard Deviation, *ADF* Augmented Dickey-Fuller-Test, *PP* Phillips-Perron-Test

Stationarity tests are conducted including an intercept, *t*-statistics of the tests are displayed, */**/*** signal significance at the 10%/5%/1% level

Descriptive statistics as well as stationarity tests of the variables in line with the suggestion of Hasan et al. (2022) are presented in Table 1. It is obvious from the Jarque-Bera-test that all variables appear to follow a non-normal distribution. Moreover, i.e., the five asset prices as well as the effective exchange rates appear to be non-stationary in levels. Therefore, we follow the same procedure as frequently used in cryptocurrency estimations and build daily growth rates, thus e.g., asset returns, with respect to all variables.^5^ When applying this transformation, all variables appear to be stationary.

## Results

This section presents our estimation results. Since we are mainly interested in the differences between gold and the various cryptocurrencies, we follow a two-step procedure. In the first step, we estimate a bivariate system consisting of the asset prices gold and only one cryptocurrency. This leads to pairwise comparisons of the different cryptocurrencies with gold. In a second step, we estimate the model as described in Sect. 3, thus including gold and the four cryptocurrencies in one system. In order to save space in the tables, we do not show the constants of the covariance equations as they are mostly insignificant and sum the individual cross volatilities of cryptocurrencies up to one coefficient. More detailed results in both dimensions are available upon request.

Using a system estimator has the advantage that significant differences in the estimated coefficients can be detected via Wald-tests. Thus, we will use those in order to find the differences between gold and the four cryptocurrencies.

**Table 2:** System estimates with VIX

	Broad exchange rate					Narrow exchange rate				
	I	II	III	IV	V	VI	VII	VIII	IX	X
*Gold*										
Liquidity premium	−0.00 (0.00)	0.00 (0.00)	0.00 (0.00)	0.00 (0.00)	0.00 (0.00)	0.00 (0.00)	0.00 (0.00)	0.00 (0.00)	0.00 (0.00)	0.00 (0.00)
Exchange rate	−0.47*** (0.06)	−0.44*** (0.12)	−0.51*** (0.10)	−0.45*** (0.12)	−0.47*** (0.11)	−0.47*** (0.05)	−0.55*** (0.11)	−0.57*** (0.09)	−0.55*** (0.11)	−0.56*** (0.11)
Global uncertainty	0.01*** (0.00)	0.00 (0.00)	0.00 (0.00)	0.00 (0.00)	0.00 (0.00)	0.01*** (0.00)	−0.00 (0.00)	−0.00 (0.00)	−0.00 (0.00)	−0.00 (0.00)
Volatility	0.03 (0.03)	0.08** (0.04)	0.07** (0.03)	0.10** (0.05)	−0.05 (0.11)	0.03 (0.03)	0.07 (0.05)	0.07* (0.04)	0.10* (0.05)	0.09** (0.04)
Cross volatility crypto	0.03 (0.03)	−0.03 (0.03)	−0.02 (0.03)	−0.04 (0.04)	0.22* (0.12)	0.03 (0.03)	−0.01 (0.04)	−0.02 (0.03)	−0.03 (0.04)	−0.05 (0.04)
Volatility equation										
Constant	0.00*** (0.00)	0.03*** (0.01)	0.01*** (0.00)	0.02*** (0.01)	0.00*** (0.00)	0.00*** (0.00)	0.01*** (0.00)	0.01*** (0.00)	0.02*** (0.01)	0.03*** (0.01)
ARCH(-1)	0.17*** (0.01)	0.31*** (0.02)	0.25*** (0.02)	0.29*** (0.02)	0.04** (0.02)	0.17*** (0.01)	0.22*** (0.02)	0.23*** (0.02)	0.26*** (0.02)	0.29*** (0.02)
GARCH(-1)	0.98*** (0.00)	0.94*** (0.01)	0.96*** (0.01)	0.95*** (0.01)	1.00*** (0.00)	0.98*** (0.00)	0.97*** (0.01)	0.97*** (0.01)	0.95*** (0.01)	0.94*** (0.09)
*Bitcoin*										
Liquidity premium	−0.00 (0.00)				−0.00 (0.00)	−0.00 (0.00)				−0.00 (0.00)
Exchange rate	−0.55 (0.34)				−0.93 (0.58)	−0.44 (0.29)				−0.56 (0.57)
Global uncertainty	−0.04*** (0.01)				−0.05*** (0.02)	−0.04*** (0.01)				−0.06*** (0.02)
Volatility	0.01** (0.00)				0.03 (0.04)	0.01** (0.00)				0.03 (0.02)
Cross volatility gold	0.14 (0.13)				0.66 (0.68)	0.14 (0.13)				0.20 (0.18)
Cross volatility crypto					−0.04 (0.04)					−0.02 (0.03)
Volatility equation										
Constant	0.87*** (0.10)				1.04*** (0.15)	0.87*** (0.10)				2.52*** (0.72)
ARCH(-1)	0.36*** (0.01)				0.22*** (0.01)	0.36*** (0.01)				0.31*** (0.04)
GARCH(-1)	0.92*** (0.01)				0.95*** (0.01)	0.92*** (0.01)				0.89*** (0.03)
*Ethereum*										
Liquidity premium		−0.00 (0.00)			−0.00 (0.00)		−0.00 (0.00)			−0.00 (0.00)
Exchange rate		−1.95** (0.76)			−1.88** (0.76)		−1.06 (0.75)			−1.06 (0.76)
Global uncertainty		−0.06** (0.03)			−0.07*** (0.03)		−0.08*** (0.03)			−0.08*** (0.03)
Volatility		0.01 (0.01)			−0.02 (0.04)		0.01 (0.01)			0.02 (0.01)
Cross volatility gold		0.25 (0.18)			1.69** (0.78)		0.31 (0.22)			0.29 (0.19)
Cross volatility crypto					−0.01 (0.06)					−0.04 (0.04)
Volatility equation										
Constant		3.82*** (1.01)			2.26*** (0.39)		3.84*** (1.03)			3.86*** (1.14)
ARCH(-1)		0.29*** (0.02)			0.22*** (0.02)		0.30*** (0.03)			0.30*** (0.04)
GARCH(-1)		0.90*** (0.02)			0.95*** (0.01)		0.90*** (0.02)			0.90*** (0.02)
*Bitcoin Cash*										
Liquidity premium			−0.00 (0.00)		0.00 (0.00)			−0.00 (0.00)		0.00 (0.00)
Exchange rate			−1.79** (0.83)		−2.35** (0.97)			−1.04 (0.81)		−1.65* (0.96)
Global uncertainty			−0.08*** (0.03)		−0.10*** (0.03)			−0.09*** (0.03)		−0.11*** (0.03)
Volatility			−0.00 (0.00)		−0.01 (0.01)			4.07** (0.04)		0.13 (0.72)
Cross volatility gold			0.39* (0.23)		0.88 (0.72)			0.46* (0.24)		0.54** (0.23)
Cross volatility crypto					0.00 (0.03)					0.04 (0.03)
Volatility equation										
Constant			3.90*** (0.62)		3.21*** (0.33)			4.28*** (0.68)		4.20*** (0.78)
ARCH(-1)			0.29*** (0.02)		0.37*** (0.02)			0.30*** (0.02)		0.32*** (0.02)
GARCH(-1)			0.93*** (0.01)		0.92*** (0.01)			0.92*** (0.01)		0.91*** (0.01)
*Litecoin*										
Liquidity premium				−0.00** (0.00)	−0.00** (0.00)				−0.00* (0.00)	−0.00** (0.00)
Exchange rate				−2.56*** (0.80)	−2.52*** (0.80)				−1.42* (0.79)	−1.47* (0.79)
Global uncertainty				−0.06** (0.03)	−0.06** (0.03)				−0.08*** (0.03)	−0.08*** (0.03)
Volatility				0.00 (0.01)	0.02 (0.03)				0.00 (0.01)	0.01 (0.01)
Cross volatility gold				0.32 (0.22)	0.70 (0.65)				0.32 (0.22)	0.42* (0.23)
Cross volatility crypto					−0.01 (0.06)					0.01 (0.04)
Volatility equation										
Constant				1.54*** (0.36)	1.54*** (0.20)				1.68*** (0.40)	5.18*** (1.51)
ARCH(-1)				0.22*** (0.02)	0.24*** (0.01)				0.23*** (0.02)	0.28*** (0.03)
GARCH(-1)				0.96*** (0.01)	0.96*** (0.00)				0.95*** (0.01)	0.89*** (0.03)
log-likelihood	−7363.11	−3129.69	−4082.02	−3162.20	−8788.39	−7356.84	−3124.04	−4082.50	−3163.49	−9342.59
N	1822	702	890	702	702	1822	702	890	702	702

*Notes:* All variables in daily percentage changes. Dependent variable is the gold price or the price of cryptocurrencies. Liquidity premium = bid-ask-spread, exchange rate = broad (narrow) effective exchange rate towards 58 (24) other economies, global uncertainty = VIX, volatility = GARCH, cross-volatility = GARCH covariance. Standard errors in parentheses. Significance level of 1%, 5% and 10% is denoted by ***, ** and *

The results when using the VIX as a global uncertainty indicator are presented in Table 2, while the corresponding Wald-tests for statistically different coefficients are shown in Table 3. The tables show two sets of estimates, one using the broad effective exchange rate and one using the narrow aggregate. We can draw several conclusions from these results: First, the liquidity premium seems to be hardly relevant for either gold or cryptocurrencies. The only exception is Litecoin, where indeed the expected significant negative coefficient can be found, while for all other assets the results are insignificant. Therefore, it does not come as a surprise that the only significant differences between gold and a cryptocurrency with respect to the liquidity premium can be found vis-a-vis Litecoin, thus for Litecoin, the response to liquidity issues is significantly more pronounced than for gold.

**Table 3:** Differences between gold and cryptocurrencies with VIX

	Broad exchange rate					Narrow exchange rate				
	I	II	III	IV	V	VI	VII	VIII	IX	X
*Gold versus Bitcoin*										
Liquidity premium	0.00 (0.97)				0.50 (0.48)	0.00 (0.98)				0.87 (0.35)
Exchange rate	0.05 (0.81)				0.63 (0.42)	0.00 (0.93)				0.00 (0.99)
Global uncertainty	15.13*** (0.00)				7.17*** (0.01)	15.74*** (0.00)				7.86*** (0.01)
Volatility	0.63 (0.43)				0.43 (0.51)	0.56 (0.45)				1.63 (0.20)
Cross volatility gold	0.73 (0.39)				0.35 (0.56)	0.64 (0.42)				0.78 (0.38)
Cross volatility crypto					1.13 (0.29)					0.69 (0.41)
*Gold versus Ethereum*										
Liquidity premium		0.92 (0.34)			0.54 (0.46)		0.93 (0.33)			0.90 (0.34)
Exchange rate		3.81* (0.05)			3.35* (0.07)		0.47 (0.50)			0.44 (0.51)
Global uncertainty		9.97*** (0.00)			6.98*** (0.01)		8.38*** (0.00)			8.65*** (0.00)
Volatility		3.35* (0.07)			0.04 (0.84)		1.92 (0.17)			2.47 (0.12)
Cross volatility gold		2.12 (0.15)			1.66 (0.20)		2.10 (0.15)			1.58 (0.21)
Cross volatility crypto					1.25 (0.26)					0.31 (0.58)
*Gold versus Bitcoin Cash*										
Liquidity premium			0.20 (0.66)		0.10 (0.75)			0.28 (0.60)		0.07 (0.79)
Exchange rate			2.32 (0.13)		3.73* (0.05)			0.33 (0.56)		1.28 (0.26)
Global uncertainty			8.12*** (0.00)		8.69*** (0.00)			10.02*** (0.00)		10.26*** (0.00)
Volatility			4.07** (0.04)		0.13 (0.72)			3.41* (0.06)		1.63 (0.20)
Cross volatility gold			3.13* (0.08)		0.13 (0.71)			3.95** (0.05)		4.60** (0.03)
Cross volatility crypto					1.41 (0.23)					3.09* (0.08)
*Gold versus Litecoin*										
Liquidity premium				2.95* (0.09)	2.70 (0.10)				3.02* (0.08)	3.10* (0.08)
Exchange rate				6.79*** (0.01)	6.38** (0.01)				1.18 (0.28)	1.30 (0.25)
Global uncertainty				5.19** (0.02)	5.70** (0.02)				7.70*** (0.01)	8.11*** (0.00)
Volatility				3.66* (0.06)	0.36 (0.55)				3.08* (0.08)	2.75* (0.10)
Cross volatility gold				2.67 (0.10)	0.02 (0.88)				2.47 (0.11)	2.54 (0.11)
Cross volatility crypto					1.32 (0.25)					1.47 (0.22)

*Notes:* Wald-tests for coefficient equality based on the estimates of the previous table. Chi-square test statistic, *p*-value in parentheses. A significance level of 1%, 5% and 10% is denoted by ***, ** and *

Second, the response to the effective exchange rate is for all assets found to be negative as expected, and it turns out to be mainly significant. However, the coefficients differ on the one hand with respect to the different assets and on the other hand between the broad and narrow effective exchange rate. While the coefficients are of almost the same size with about $-0.5$ for gold in all specifications, the response coefficients turn out to be higher for Ethereum, Bitcoin Cash and Litecoin with levels exceeding −1. Even more important, the estimates for those three cryptocurrencies tend to be higher when using the broad effective exchange rate instead of the narrow aggregate. This leads to the result that only for the broad aggregate, we identify significant differences between gold and the three cryptocurrencies, i.e., that those three react stronger to changes in the exchange rate than gold. The result that those three cryptocurrencies show a stronger response to the broad effective exchange rate is reasonable since the difference between the broad and narrow aggregate includes i.e., developing and transition countries with overall a less credible central bank than developed countries forming the narrow index. As the domestic central bank is less credible, private cryptocurrencies are presumably a more relevant alternative in those countries than in developed countries.

Third, there are significant differences between all four cryptocurrencies and gold regarding the impact of global uncertainty. While for gold the response is (if anything) significantly positive underlining the role of gold as a safe-haven asset, the coefficients turn out to be significantly negative for all cryptocurrencies in all specifications. Thus, the cryptocurrencies cannot be viewed as a safe-haven in times of financial stress. Thus, our results are in line with those Akyildirim et al. (2020), Corbet et al. (2020) or Demir et al. (2020). It does thus not come as a surprise that compared to gold, the difference with respect to global uncertainty is significantly lower for all four cryptocurrencies.

Fourth, concerning volatility, investors indeed tend to demand a premium. This holds for either gold and cryptocurrencies. Quite astonishingly, the premium tends to be higher for gold than for Ethereum, Bitcoin Cash and Litecoin, even though only in a few of those specifications the difference is significant. However, this effect is partly offset by the higher response coefficients if gold and the cryptocurrencies move in tandem, even though only for Bitcoin Cash these differences turn out to be significant.

Finally, if the volatility among cryptocurrencies moves in tandem, there is no additional liquidity premium demanded, possibly because risks with respect to the different cryptocurrencies is viewed as being equal. With this our results support the findings of Corbet et al. (2018) Katsiampa (2018) or Andrada-Felix et al. (2020)

**Table 4:** System estimates with BBB-government bond spread

	Broad exchange rate					Narrow exchange rate				
	I	II	III	IV	V	VI	VII	VIII	IX	X
*Gold*										
Liquidity premium	−0.00 (0.00)	0.00 (0.00)	0.00 (0.00)	0.00 (0.00)	0.00 (0.00)	−0.00 (0.00)	0.00 (0.00)	0.00 (0.00)	0.00 (0.00)	0.00 (0.00)
Exchange rate	−0.53*** (0.06)	−0.49*** (0.13)	−0.57*** (0.10)	−0.50*** (0.13)	−0.53*** (0.13)	−0.48*** (0.05)	−0.54*** (0.12)	−0.57*** (0.09)	−0.54*** (0.12)	−0.59*** (0.12)
Global uncertainty	0.08*** (0.01)	0.03 (0.02)	0.04** (0.02)	0.03 (0.02)	0.03 (0.02)	0.06*** (0.00)	0.02 (0.02)	0.02 (0.02)	0.01 (0.02)	0.02 (0.02)
Volatility	0.07*** (0.03)	0.12** (0.04)	0.10*** (0.03)	0.15*** (0.04)	−0.06 (0.13)	0.06** (0.03)	0.11*** (0.04)	0.10*** (0.03)	0.14*** (0.04)	−0.06 (0.13)
Cross volatility crypto	−0.05** (0.02)	−0.08*** (0.03)	−0.08 (0.02)	−0.11*** (0.04)	0.23 (0.16)	−0.03 (0.02)	−0.06** (0.03)	−0.07*** (0.02)	−0.09** (0.04)	0.32* (0.17)
Volatility equation										
Constant	0.01*** (0.00)	0.02*** (0.01)	0.02*** (0.00)	0.03*** (0.01)	0.00*** (0.00)	0.02*** (0.00)	0.02*** (0.01)	0.01*** (0.00)	0.03*** (0.01)	0.00*** (0.01)
ARCH(−1)	0.23*** (0.01)	0.30*** (0.02)	0.29*** (0.02)	0.33*** (0.02)	0.02 (0.02)	0.27*** (0.02)	0.28*** (0.02)	0.23*** (0.02)	0.31*** (0.02)	0.02 (0.02)
GARCH(−1)	0.97*** (0.00)	0.94*** (0.01)	0.94*** (0.01)	0.93*** (0.01)	1.00*** (0.00)	0.95*** (0.01)	0.95*** (0.01)	0.97*** (0.01)	0.94*** (0.01)	1.00*** (0.00)
*Bitcoin*										
Liquidity premium	−0.00 (0.00)				−0.00 (0.00)	−0.00 (0.00)				−0.00 (0.00)
Exchange rate	−0.37 (0.36)				−0.63 (0.62)	−0.35 (0.30)				−0.20 (0.58)
Global uncertainty	−0.28*** (0.08)				−0.22** (0.10)	−0.30*** (0.08)				−0.26*** (0.09)
Volatility	0.01** (0.00)				0.02 (0.04)	0.01** (0.00)				0.01 (0.04)
Cross volatility gold	0.11 (0.11)				0.30 (0.75)	0.13 (0.12)				0.28 (0.77)
Cross volatility crypto					−0.01 (0.04)					−0.01 (0.04)
Volatility equation										
Constant	0.91*** (0.10)				1.04*** (0.13)	0.90*** (0.10)				1.01*** (0.13)
ARCH(−1)	0.36*** (0.01)				0.24*** (0.02)	0.36*** (0.01)				0.24*** (0.02)
GARCH(−1)	0.92*** (0.01)				0.95*** (0.01)	0.92*** (0.01)				0.95*** (0.01)
*Ethereum*										
Liquidity premium		−0.00 (0.00)			−0.00 (0.00)		−0.00 (0.00)			−0.00 (0.00)
Exchange rate		−1.46* (0.82)			−1.42* (0.82)		−0.65 (0.77)			−0.52 (0.76)
Global uncertainty		−0.34*** (0.013)			−0.31** (0.13)		−0.42*** (0.12)			−0.39*** (0.12)
Volatility		0.01 (0.01)			−0.04 (0.04)		0.01 (0.01)			−0.04 (0.04)
Cross volatility gold		0.27 (0.18)			1.34 (0.84)		0.29 (0.18)			1.35 (0.84)
Cross volatility crypto					0.02 (0.06)					0.02 (0.06)
Volatility equation										
Constant		4.33*** (1.17)			2.28*** (0.38)		4.38*** (1.16)			2.27*** (0.36)
ARCH(−1)		0.30*** (0.02)			0.23*** (0.02)		0.28*** (0.02)			0.23*** (0.02)
GARCH(−1)		0.89*** (0.02)			0.95*** (0.01)		0.89*** (0.02)			0.95*** (0.00)
*Bitcoin Cash*										
Liquidity premium			−0.00 (0.00)		−0.00 (0.00)			−0.00 (0.00)		−0.00 (0.00)
Exchange rate			−1.69** (0.88)		−2.35** (1.05)			−0.93 (0.83)		−0.91 (0.97)
Global uncertainty			−0.31** (0.15)		−0.20 (0.16)			−0.41*** (0.14)		−0.32** (0.15)
Volatility			0.00 (0.00)		−0.01 (0.01)			−0.00 (0.00)		−0.01 (0.01)
Cross volatility gold			0.43** (0.20)		0.61 (0.83)			0.53*** (0.20)		0.63 (0.85)
Cross volatility crypto					0.01 (0.03)					0.01 (0.03)
Volatility equation										
Constant			6.35*** (0.99)		3.47*** (0.32)			6.81*** (1.10)		3.57*** (0.33)
ARCH(−1)			0.32*** (0.02)		0.36*** (0.02)			0.27*** (0.02)		0.36*** (0.02)
GARCH(−1)			0.89*** (0.01)		0.95*** (0.01)			0.88*** (0.02)		0.92*** (0.01)
*Litecoin*										
Liquidity premium				−0.00* (0.00)	−0.00** (0.00)				−0.00* (0.00)	−0.00** (0.00)
Exchange rate				−2.17** (0.87)	−2.16*** (0.87)				−1.02 (0.81)	−0.89 (0.81)
Global uncertainty				−0.31** (0.13)	−0.27** (0.14)				−0.42*** (0.13)	−0.38*** (0.13)
Volatility				0.00 (0.01)	0.01 (0.03)				0.00 (0.01)	0.01 (0.03)
Cross volatility gold				0.39* (0.21)	0.62 (0.73)				0.42** (0.21)	0.56 (0.73)
Cross volatility crypto					0.00 (0.05)					0.01 (0.05)
Volatility equation										
Constant				2.02*** (0.52)	1.59*** (0.20)				2.36*** (0.62)	2.16*** (0.22)
ARCH(−1)				0.22*** (0.02)	0.25*** (0.01)				0.23*** (0.02)	0.25*** (0.01)
GARCH(−1)				0.95*** (0.01)	0.96*** (0.00)				0.95*** (0.01)	0.96*** (0.00)
log-likelihood	−7334.16	−3114.09	−4068.74	−3152.24	−8786.44	−7336.75	−3116.28	−4074.44	−3156.13	−8785.89
N	1822	702	890	702	702	1822	702	890	702	702

*Notes:* All variables in daily percentage changes. Dependent variable is the gold price or the price of cyrptocurrencies. Liquidity premium = bid-ask-spread, exchange rate = broad (narrow) effective exchange rate towards 58 (24) other economies, global uncertainty = BBB government bond spread, volatility = GARCH, cross-volatility = GARCH covariance. Standard errors in parentheses. Significance level of 1%, 5% and 10% is denoted by ***, ** and *

**Table 5:** Differences between gold and cryptocurrencies with BBB-government bond spread

	Broad exchange rate					Narrow exchange rate				
	I	II	III	IV	V	VI	VII	VIII	IX	X
*Gold versus Bitcoin*										
Liquidity premium	0.10 (0.76)				0.42 (0.51)	0.03 (0.87)				0.50 (0.48)
Exchange rate	0.17 (0.68)				0.02 (0.88)	0.19 (0.66)				0.42 (0.52)
Global uncertainty	18.57*** (0.00)				6.51** (0.01)	19.29*** (0.00)				8.67*** (0.00)
Volatility	6.11** (0.01)				0.29 (0.59)	4.00** (0.05)				0.26 (0.61)
Cross volatility gold	2.09 (0.15)				0.10 (0.75)	1.93 (0.16)				0.28 (0.59)
Cross volatility crypto					0.57 (0.45)					1.00 (0.32)
*Gold versus Ethereum*										
Liquidity premium		1.03 (0.31)			0.49 (0.48)		1.10 (0.29)			0.58 (0.45)
Exchange rate		1.38 (0.24)			1.15 (0.28)		0.02 (0.89)			0.01 (0.93)
Global uncertainty		8.09*** (0.00)			6.96*** (0.01)		12.76*** (0.00)			11.05*** (0.00)
Volatility		8.40*** (0.00)			0.01 (0.90)		6.08** (0.01)			0.01 (0.91)
Cross volatility gold		3.75* (0.05)			0.55 (0.46)		3.75* (0.05)			0.29 (0.59)
Cross volatility crypto					0.67 (0.41)					1.10 (0.29)
*Gold versus Bitcoin Cash*										
Liquidity premium			0.15 (0.70)		0.19 (0.66)			0.23 (0.63)		0.24 (0.62)
Exchange rate			1.59 (0.21)		2.96* (0.09)			0.19 (0.66)		0.11 (0.74)
Global uncertainty			5.17** (0.02)		1.91 (0.17)			8.66*** (0.00)		4.68** (0.03)
Volatility			10.27*** (0.00)		0.14 (0.71)			8.60*** (0.00)		0.15 (0.70)
Cross volatility gold			6.85*** (0.01)		0.00 (0.99)			8.71*** (0.00)		0.03 (0.87)
Cross volatility crypto					0.65 (0.42)					1.07 (0.30)
*Gold versus Litecoin*										
Liquidity premium				2.90* (0.09)	2.44 (0.12)				3.06* (0.08)	2.58 (0.11)
Exchange rate				3.65* (0.06)	3.45* (0.06)				0.34 (0.56)	0.14 (0.71)
Global uncertainty				6.16** (0.01)	4.81** (0.03)				11.73*** (0.00)	9.44*** (0.00)
Volatility				11.47*** (0.00)	0.27 (0.60)				8.73*** (0.00)	0.27 (0.61)
Cross volatility gold				5.40** (0.02)	0.00 (0.98)				5.64** (0.02)	0.07 (0.79)
Cross volatility crypto					0.62 (0.43)					1.06 (0.30)

*Notes:* Wald-tests for coefficient equality based on the estimates of the previous table. Chi-square test statistic, p-value in parentheses. A significance level of 1%, 5% and 10% is denoted by ***, ** and *

When using the corporate BBB government bond spread instead of the VIX as indicator for global uncertainty, the results are mainly reinforced (see Tables 4 and 5), i.e., with respect to the liquidity premium, exchange rate and liquidity premia. Although the coefficient size with respect to global uncertainty differs from the previous estimate, the very same conclusion can be drawn now, meaning that gold exhibits a safe-haven status while the four cryptocurrencies do not.

## Conclusions

In this article, we have estimated a novel system-GARCH-in-mean for gold and four cryptocurrencies and identified significant differences between both types. To the best of our knowledge we are the first to include a measure of liquidity and exchange rates into the estimation, even though we found that liquidity premia play less of a role in all assets. The negative response to exchange rate changes is more pronounced for cryptocurrencies, i.e., if developing countries are included in the exchange rate. Volatility premia tend to exist for all assets which is in line with the findings of Corbet et al. (2018); Katsiampa (2017, 2019) or Andrada-Felix et al. (2020). However, we cannot verify the volatility spillovers of the just mentioned studies between cryptocurrencies. This may be the result of the different sample period or the different set of cryptocurrencies. Most importantly, cryptocurrencies are different from gold when it comes to global uncertainty. While gold is seen as a safe-haven in times of rising stress, the reverse is true with respect to all four cryptocurrencies, thus our results are in line with those of Baur and Dimpfl (2018). Please note however, that those results may change in the future, as new data becomes available, like in other time-series studies.

The conclusion that can be drawn from our analysis is that cryptocurrencies differ among each other but not in all dimensions. While we have shown that there are differences in cryptocurrencies, at least with respect to liquidity, volatility and the exchange rate, they do not differ for global uncertainty. Thus, all of them do not fulfill one major property of a currency or gold, which is being a store of value. This being said, cryptocurrencies have to be seen as speculative assets. So there could be the need to regulate those assets in order to prevent financial crisis resulting from them. But we have also seen, that regulation needs to be coordinated at a global level as the cryptocurrencies are not bound to specific countries. This being said, not only developed countries need to find a coordinated approach for regulations but also developing countries need to be incorporated.

## Footnotes

1 Of course the role of volatility in cryptocurrencies has also been investigated without the focus on interconnectedness of the various cryptocurrencies. See e.g., Katsiampa (2017), Baur and Dimpfl (2018), Chaim and Laurini (2018, 2019) and Troster et al. (2019).

2 The 24 economies covered in the narrow exchange rate towards the US-dollar are: Australia, Austria, Belgium, Canada, Chinese Taipei, Denmark, Finland, France, Germany, Greece, Hong Kong, Ireland, Italy, Japan, Korea, Netherlands, New Zealand, Norway, Portugal, Singapore, Spain, Sweden, Switzerland and United Kingdom.

3 The additional 34 economies covered in the broad effective exchange rate are: Algeria, Argentina, Brazil, Bulgaria, Chile, China, Colombia, Croatia, Cyprus, Czech Republic, Estonia, Hungary, Iceland, India, Indonesia, Israel, Latvia, Lithuania, Luxembourg, Malaysia, Malta, Mexico, Peru, Philippines, Poland, Romania, Russia, Saudi Arabia, Slovak Republic, Slovenia, South Africa, Thailand, Turkey and United Arab Emirates.

4 Other studies using the US corporate BBB government bond spread as a measure of global uncertainty are Dötz and Fischer (2010), Bernoth and Erdogan (2012), Klose and Weigert (2014) or Klose (2021).

5 See e.g., Ardia et al. (2019), Liu and Serletis (2019), Akyildirim et al. (2020), Corbet et al. (2020b), Liu and Tsyvinski (2021), Hassan et al. (2021).
